# Aloperine Relieves Type 2 Diabetes Mellitus *via* Enhancing GLUT4 Expression and Translocation

**DOI:** 10.3389/fphar.2020.561956

**Published:** 2021-01-25

**Authors:** Guanjun Song, Yun Huang, Mingrui Xiong, Ziwei Yang, Qinghua Liu, Jinhua Shen, Ping Zhao, Xinzhou Yang

**Affiliations:** ^1^ Institute for Medical Biology and Hubei Provincial Key Laboratory for Protection and Application of Special Plants in the Wuling Area of China, College of Life Sciences, South-Central University for Nationalities, Wuhan, China; ^2^ School of Pharmaceutical Sciences, South-Central University for Nationalities, Wuhan, China

**Keywords:** type 2 diabetes mellitus, glucose transporter 4, Ca^2+^, L6 cells, protein kinase C, Akt

## Abstract

Aloperine (ALO), a quinolizidine alkaloid isolated from *Sophora alopecuroides* L. used in the traditional Uygur medicine, induced a significant increase in cellular glucose uptake of L6 cells, suggesting it has the potential to relieve hyperglycemia. Therefore, we investigated the effects of ALO on type 2 diabetes mellitus (T2DM) through *in vitro* and *in vivo* studies. The translocation of glucose transporter 4 (GLUT4) and changes in intracellular Ca^2+^ levels were real-time monitored in L6 cells using a laser scanning confocal microscope and related protein kinase inhibitors were used to explore the mechanism of action of ALO. Furthermore, high fat diet combined with low-dose streptozotocin (STZ) was used to induce T2DM in rats, and ALO was given to the stomach of T2DM rats for 4 weeks. *In vitro* results showed that ALO-induced enhancement of GLUT4 expression and translocation were mediated by G protein-PLC-PKC and PI3K/Akt pathways and ALO-enhanced intracellular Ca^2+^ was involved in activating PKC *via* G protein-PLC-IP_3_R-Ca^2+^ pathway, resulting in promoted GLUT4 plasma membrane fusion and subsequent glucose uptake. ALO treatment effectively ameliorated hyperglycemia, glucose intolerance, insulin resistance and dyslipidemia, alleviated hepatic steatosis, protected pancreatic islet function and activated GLUT4 expression in insulin target tissues of T2DM rats. These findings demonstrated that ALO deserves attention as a potential hypoglycemic agent.

## Introduction

Diabetes mellitus is an endocrinal and metabolic disease, caused by the inability of pancreas to produce required insulin and/or an inability of the body to effectively respond to insulin (Lauritano and Ianora, 2016). More than 90 percent of diabetic cases were type 2 diabetes mellitus (T2DM) (Reimann et al., 2009). Insulin resistance is the typical characteristic of T2DM, a condition in which the targeted cells fail to respond to the hormone insulin stimulation (Kadowaki, 2000). Dysfunction of glucose transporters, glycogen synthesis, and glycogen oxidation can result in insulin resistance in the muscle tissue.

Glucose transporter 4 (GLUT4) is the main carrier of glucose transport. Selective disruption of GLUT4 expression in muscle induce global insulin resistance (Zisman et al., 2000). GLUT4 heterozygous knockout mice developed insulin signaling defects in adipocytes with the progression of whole body insulin resistance and diabetes (Li et al., 2000). GLUT4 is the main protein that transports blood glucose into the cells of muscle and fat tissue, which therefore, has been thought as a therapeutic target for pharmacological intervention strategies to control diabetic hyperglycemia (Carvalho et al., 2005; Morgan et al., 2011).

As an important second messenger of organisms, Ca^2+^ plays a crucial role in the transport of GLUT4 to the plasma membrane. Ryanodine receptors (RyRs) and inositol 1,4,5-trisphosphate receptors (IP3Rs) are Ca^2+^ release channel sonthe endo/sarcoplasmic reticulum (ER/SR) (Santulli et al., 2017). Studies have shown that insulin-induced Ca^2+^ increase mediated by RyR1 and IP3R is a component of insulin signaling of GLUT4 translocation in skeletal muscle canals (Contreras-Ferrat et al., 2014). RyR channel agonist stimulated GLUT4*myc* translocation and insulin stimulated RyR1-mediated Ca^2+^ release by promoting RyR1 Sglutathionylation (Contreras-Ferrat et al., 2014; Li et al., 2014). The effect of ALO on intracellular Ca^2+^ concentration was also investigated in this study.

**FIGURE 1:**
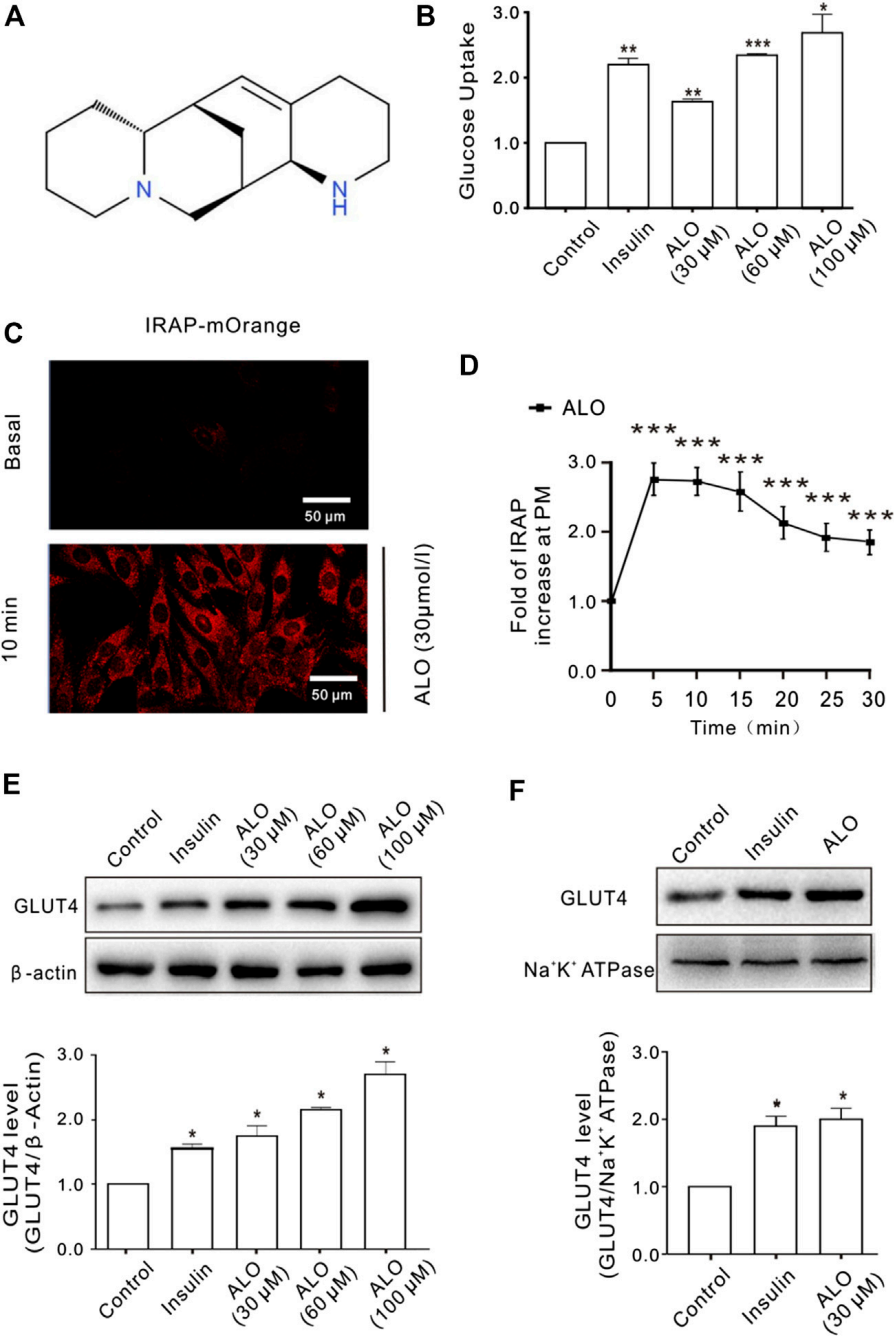
ALO increased glucose uptake and enhanced GLUT4 translocation and expression in L6 cells. **(A)** Chemical structure of ALO. **(B)** Glucose uptake was measured by 2-NBDG assay. Insulin and ALO (30, 60, and 100 μM) increased glucose uptake, respectively. *p < 0.05; **p < 0.01; ***p < 0.001. **(C)** IRAP fluorescence in L6 cells was measured by confocal microscope. Following the addition of ALO, IRAP fluorescence intensity at the PM was increased significantly at 10 min. Scale bar: 50 μm. **(D)** Time course of the change in fluorescence induced by ALO. Fluorescence was normalized to the value prior to ALO addition. ***p < 0.001. **(E)** Insulin and ALO (30, 60, and 100 μM) induced an increase in GLUT4 protein level in L6 cells. *p < 0.05. **(F)** ALO (30 μM) induced an increase in GLUT4 protein level in plasma membrane fraction of L6 cells. *p < 0.05 [in **(B,E–F)** Data are means ± SEM (*n* = 3); in **(C,D)** Data are means ± SEM (*n* = 30)].

Natural botanical medicine is a new trend in modern clinical medicine. It has a long history as an alternative treatment for T2DM, with good curative effects and few side effects. Traditional Chinese medicine and its natural products have shown great potential in preventing and treating T2DM (Li et al., 2004). *Sophora alopecuroides* L., a traditional Uygur medicinal plant mainly used in Xinjiang Autonomous region of China to protect against heat and dampness, killing insects and relieving pain (Huang and Li, 2002). Aloperine (ALO), a quinolizidine alkaloid, extracted from the roots and leaves of *S. alopecuroides* L (Figure 1A). possessed promising biological effects including antiviral, anti-tumor, anti-inflammatory, neuroprotective and antinociceptive activities (Fu et al., 2017; Ling et al., 2018; Zhang et al., 2018). However, its effect for diabetes has not been explored yet. We discovered that ALO had significant effects in promoting glucose uptake *via* targeting GLUT4 *in vitro.* Thus, further *in vitro* and *in vivo* experiments were conducted to examine the antidiabetic effects and molecular mechanisms of ALO.

Rat skeletal muscle L6 cell is a well-established model for studying glucose uptake process and GLUT4 trafficking since skeletal muscle is one of the insulin target tissues involved in regulating glucose homeostasis and GLUT4 plays a vital role in the process. In order to gain more insight into the mechanism of action of ALO, protein kinase inhibitors associated with GLUT4 trafficking and expression were used to investigate whether they were involved in mediating GLUT4 activity. Besides, we induced the T2DM rat by high-fat diet (HFD) combined with low dose of streptozotocin (STZ) injection, which closely mimicked metabolic characteristics of humans T2DM (Reed et al., 2000). The effects of ALO on glucose and lipid metabolisms, histopathology changes and protein expression in insulin target tissues were investigated to clarify its beneficial effects for the treatment of T2DM.

## Materials and Methods

### Reagents

Compound C was purchased from Calbiochem (San Diego, CA, United States). Wortmannin was purchased from Selleckchem (Houston, TX, United States). Gö6983 was purchased from EMD Millipore (Billerica, MA). Fluo-4 AM was purchased from Invitrogen (Camarillo, CA, United States). Pertussis Toxin (PTX) and Gallein were purchased from Tocris Bioscience (Bristol, United Kingdom). Ryanodine was purchased from Cayman Chemical (Ann Arbor, MI, United States). 1,2-Bis (2-aminophenoxy) ethane-N,N,N′,N′-tetraacetic acid (BAPTA-AM, Caffeine, a chelator of Ca^2+^), U73122 and 2-APB were purchased from Sigma (St. Louis, MO, United States). GLUT4 antibody (#2213), Akt antibody (#9272), phospho-Akt (Ser473) (193H12) antibody (#4058) and phospho-PKC (pan) (Thr410) antibody (#2060) were purchased from Cell Signaling Technology (Beverly, MA, United States). The Anti-c-myc mouse monoclonal antibody (#M10117) and FITC antibody (#M10422) were both purchased from TransGen Biotech (Beijing, China).

### Isolation and Purification of Aloperine

In the previous study, the method of plant separation and purification was described (Shu et al., 2015; Chen et al., 2018). The aerial parts of *S. alopecuroides* L. were purchased from Herb Market, Xining City, Qinghai Province in August 2015 and identified by Prof. Dingrong Wan, School of Pharmaceutical Sciences, South-Central University for Nationalities (SCUN), Wuhan, China. A voucher specimens of *S. alopecuroides* L*.* was numbered (No. SC0859) and has been deposited in the Herbarium of School of Pharmaceutical Sciences, SCUN. Air dried aerial parts of *S. alopecuroides* L. (10°kg) were ground into powder and extracted using 85% EtOH (4 × 30 L, 4°days each). After evaporation of the collected solution, the residue was acidified with diluted HCl (5%) to a pH of two and partitioned between petroleum ether (60–90°C, 6 × 2.5 L) and the acid water layer. The aqueous part was basified with aqueous NH_3_ to a pH of 10 and extracted with ethyl acetate to afford 135 g of crude alkaloid. A part of the crude alkaloid (125 g) was subjected to column chromatography over silica gel (300–400 mesh, 4.5°kg) and eluted with the isocratic gradient solvent system of CH_2_Cl_2_, methanol, and diethylamide (100: 10: 2) to yield six fractions (F1−F6) (F1, 4.5°L; F2, 6.8°L; F3, 8.5°L; F4, 8.8°L; F5, 7.6 L). After evaporation under vacuum, crude aloperine (33 g) was obtained from F3 and then dissolved in methanol (150 ml) at 50°C. The ALO solution was kept in refrigerator at 4°C to recrystallize and afford 19 g of pure ALO. ^1^H, ^13^C NMR and ESIMS data were obtained to support the identification of aloperine (Supplementary Figure S1). The ALO was analyzed by high-performance liquid chromatography (HPLC) on a Waters 1525 HPLC instrument by a single injection of 20 μl with an analytical C18 HPLC column (Betasil 150 × 4.6 mm, 5 μm) detected at 254 nm. Gradient conditions were set as 90% H2O (0.1% TFA) + 10% MeCN (0.1% TFA) → 100% MeCN (0.1% TFA), 20 min, 100% MeCN (0.1% TFA), 25 min. Flow rate was set at 1.0 ml/min. The purity of ALO was above 98% as detected by HPLC and UV spectrum analysis.

### Plasmid and Cell Line Construction

pIRAP-mOrange cDNAs were inserted into the pQCXIP plasmid. The retrovirus was prepared by transfecting pQCXIP-IRAP-mOrange, vesicular stomatitis virus G (VSVG), and PHIT60 (include MuLV structural genes, namely gag and pol) at a ratio of 2: 1: 1 through Lipofectamine 2000 (Invitrogen, CA, United States) into Plat E cells (Morita et al., 2000). Forty-eight hour later, the cultural supernatant was collected and viruses were concentrated by super-centrifugation (at 50,000 × *g*, 30 min). L6 cells (presented by Professor Pingsheng Liu, Institute of Biophysics, the Chinese Academy of Science, Beijing) were infected with freshly prepared viruses at the exponential growth phase. Polybrene (8 μg/ml) was used to improve the efficiency of virus infection. L6 Cells with fluorescence were isolated by fluorescence activated cell sorter (FACS) and seeded into 96-well plates. Finally, the clone with the highest fluorescence intensity was screened out after insulin (100 nM) treatment.

### Assays of IRAP Translocation

GLUT4 and Insulin-regulated aminopeptidase (IRAP) co-localize in GLUT4 storage vesicles (GSVs) and previous studies indicated that they are highly co-localized (Li et al., 2019). Therefore, IRAP is used as a reporter molecule for GLUT4 translocation. The stable expression of L6-IRAP-mOronge L6 cells (L6-IRAP-mOronge) was cultured in α-MEM medium with 10% fetal bovine serum and 1% antibiotic, 37°C, 5%CO_2_. L6-IRAP-mOronge was inoculated on glass covered glass slides overnight, then starved in serum-free α-MEM for 2 h. The fluorescence imaging of L6-IRAP-mOronge was performed by laser scanning confocal microscope LSM 700 (Carl Zeiss, Jena, Germany), and the dynamic displacement of irap-mrange was observed. The photos were taken after the addition of 30 μM ALO or other agents by 555 nm excitation laser every 5 min in 30 min. The fluorescence intensity of IRAP-mOrange at the PM was measured for reflecting the GLUT4 translocation as previously described (Xiong et al., 2017). To clarify the mechanism underlying the enhancement of IRAP-mOrange fluorescence intensity, the cells were incubated in the presence of 10 *µ*M Compound C (AMPK inhibitor), 100 nM Wortmannin (PI3K inhibitor), or 10 μM Gö6983 (PKC inhibitor) for 30 min prior to treatment with ALO.

### Glucose Uptake Assay

Glucose uptake related experiments were carried out as previously described (Zhao et al., 2018). Glucose uptake in L6 cells was measured by a cell-based 2-[N-(7-nitrobenz-2-oxa-1,3-diaxol-4-yl)amino]-2-deoxyglucose (2-NBDG) glucose uptake assay kit (Cayman Chemical, United States). The L6 cells were seeded into 96-well plates with a density of 1 × 10^4^–5×10^4^ cells/well in 100 μl α-MEM medium. After 12 h incubation, the L6 cells were treated with different doses of ALO (30, 60, 100 μM) or 100 nM insulin or vehicle control dissolved in 100 μl glucose-free α-MEM medium that contained 150 μg/ml 2-NBDG. Plates were incubated at 37°C with 5% CO_2_ for 30 min. Following the treatment, the glucose uptake in the L6 cells was measured by the method described in the glucose uptake assay kit.

### Purification of L6 Cells Membrane Fraction

The steps for purification of related components of L6 cell membrane are similar to our previous experiments (Xiong et al., 2017). L6 cells were prepared and harvested after being starved in serum-free α-MEM for 2 h. The control cells and the cell that were incubated by 100 nM Insulin or 30 μM ALO were washed with cold PBS, and then supended in HES buffer (mM; 250 Sucrose, 20 HEPES, 10 EDTA, pH 7.4) containing complete protease inhibitor mixture and phosphatase inhibitors at 4°C, and lyzed by 12 passes through a 22-gauge needle followed by 12 passes through a 27-gauge needle. The lysate was then centrifuged at 500 × *g* for 10 min to remove unbroken cells and at 18,000 × *g* for 20 min to obtain the membrane fraction. All samples were subjected to SDS-PAGE analysis on 10% resolving gels. In a single experiment, each sample was loaded with the same amount of protein. The changes in expression of GLUT4 in separated proteins were detected by western blotting.

### Measurements of Intracellular Ca^2+^

Intracellular Ca^2+^ was analyzed and measured in a similar manner as previously described (Liu et al., 2009). L6 IRAP-mOrange was incubated in 2 *µ*M fluo-4 AM for 15 min at room temperature and then was superfused with fluo-4 AM-free PSS for 10 min. In different groups or situations, added the physiological saline solution (PSS) solution containing 2 mM Ca^2+^ and the PSS solution containing 0 mM Ca^2+^ into the chamber. Or added the PSS solution containing 0 mM Ca^2+^ and BAPTA to the chamber, so that the cells were in such an external fluid. The images were taken after the addition of 30 μM ALO or other agents by 488 nm excitation laser every 5 min in 30 min. The fluorescence intensity of fluo-4 AM representing the intracellular Ca^2+^ concentration was measured and analyzed by the Zen 2010 software (Carl Zeiss, Jena, Germany).

### Western Blotting Analysis

Starve L6 cells for 2 h before experiment. The L6 cells were then washed with cold PBS three times, resuspended in HES buffer supplemented with protease inhibitor cocktail (Roche, Basel, Switzerland) and phosphatase inhibitor cocktail (Selleckchem, Houston, United States) at 4°C. The L6 cells lysing was performed as described previously (Zhou et al., 2016; Zhao et al., 2018). The lysate was centrifuged at 500 × *g* for 10 min. Supernatant protein denatured in SDS sample buffer. Equal amounts of protein samples were subjected to 8% SDS-PAGE. Separated proteins were electrophoretically transferred to Polyvinylidene fluoride (PVDF) membranes, and the membranes were incubated with primary antibodies and HRP-conjugated secondary antibodies. The protein bands were detected and quantified by a ChemiDoc XRS (Bio-Rad, CA, United States).

### Animals and Treatment

The care and use of animals and all procedures involving animals were performed in accordance with the Guidelines for Animal Experiments of South-Central University for Nationalities and were approved by the university animal ethics committee (Approval Number: S08916110D). Male Sprague-Dawley (SD) rats (8 weeks old) of Specific Pathogen Free (SPF) grade were purchased from the Beijing HFK Bioscience Co., Ltd. Each three rats were fed in one cage with enough food and water under standard conditions at 23 ± 2°C, 50–60% relative humidity, and with a constant 12 h light–dark cycle. After 7 days of acclimatization, the animals were randomly assigned to receive a regular chow diet (Beijing HFK Bioscience Co., Ltd.), as the normal control group (NC, *n* = 8) or a high-fat diet (HFD; 4.73 kcal/g, 20% kcal from protein, 35% kcal from carbohydrates and 45% kcal from fat; MD12032, Medicine Co., Ltd., Yangzhou, China). After 4 weeks, the HFD rats were injected intraperitoneally with a low dose of streptozotocin (STZ, 30 mg/kg, in citrate buffer, pH 4.5; Sigma-Aldrich, St Louis, United States). Rats in the NC group were injected with the citrate buffer vehicle. Over-night fasted rats with fasting blood glucose (FBG) level more than 11.1 mmol/L at 1 week after STZ injection were chosen for experiments. Thereafter, these T2DM rats were randomly divided into four groups with eight animals each: diabetic control (DC), diabetic with low dose ALO (DAL, 20 mg/kg bw), diabetic with medium dose ALO (DAM, 40 mg/kg bw), and diabetic with high dose ALO (DAH, 80 mg/kg bw). ALO was made into a suspension with saline containing 0.5% CMC-Na. NC and DC groups were intragastric administered with the same solvent. All groups received intragastric administration once daily for four consecutive weeks.

### Body Weight, FBG Levels and OGTT

Body weight, FBG levels and oral glucose tolerance tests as previously described (Xiong et al., 2017). During the experimental period, the body weight and FBG levels of T2DM rats were measured weekly. OGTT was performed in overnight-fasted rats. Blood samples was collected from the tip of the tail at 0, 30, 60, 90, and 120 min from all groups after 2.0 g/kg glucose oral administration to measure the blood glucose level using a blood glucose meter (One Touch Ultra, Lifescan Inc., Wayne, United States) (Xiong et al., 2017). The area under the curves (AUC) generated from the data collected during the OGTT was calculated.

### Biochemical Analysis of Serum and Tissues

At the end of experiment, all rats were anesthetized with sodium pentobarbital (40 mg/kg, i. p.) and blood samples were collected from abdominal aorta. The serum was separated by centrifuging the blood samples at 3,000 × *g* for 15 min. The serum biochemical indices including total cholesterol (TC), triglycerides (TG), low density lipoprotein cholesterol (LDL-C), and high density lipoprotein cholesterol (HDL-C) were evaluated by an automatic biochemical analyzer (Hitachi 7180 + ISE, Tokyo, Japan) (Xiong et al., 2017). Serum free fatty acid (FFA), hepatic TC and TG were determined by corresponding assay kits (Jiancheng Bioengineering Institute, Nanjing, China). Serum insulin content was measured by using a rodent insulin enzyme-linked immunosorbent assay (ELISA) kit (Linco Research, St. Charles, MO).

### Histology and Immunohistochemistry

The related experiments of Histology and immunohistochemistry are similar to the previous ones (Yang et al., 2017). Parts of liver and pancreas were fixed in 4% formaldehyde and embedded in paraffin for hematoxylin–eosin (HE) staining. Frozen liver tissues embedded in OCT (optimal cutting temperature compound) were used for Oil Red O (ORO) staining. Parts of pancreas sections were incubated with mouse monoclonal anti-insulin and rabbit anti-glucagons (Sigma-Aldrich, St Louis, United States) (Wei et al., 2018). And then incubated with corresponding fluorescent-labeled secondary antibody, Alexa Fluor 594 goat anti-mouse IgG, Alexa Fluor 488 goat anti-rabbit IgG (Invitrogen, Carlsbad, United States). Nuclei were stained with DAPI. Stained sections were photographed using a Nikon Eclipse Ti-SR microscope equipped with a Nikon DS-U3 digital camera (Nikon Incorporation, Tokyo, Japan) (Yang et al., 2017).

### Tissue Extracts and Western Blotting

The procedure of tissue extraction and Western blotting is similar to that described in the previous experiment (Xiong et al., 2017). The skeletal muscle, liver and WAT (White adipose tissue) were chopped into small pieces and homogenized in ice-cold RIPA buffer (mM; 50 Tris-HCl, 150 NaCl, 1% NP-40, 0.5% sodium deoxycholate, pH 7.4) containing protease inhibitor cocktail and phosphatase inhibitor cocktail. The protein solutions were collected from the supernatant after centrifugation at 12,000 × *g*, 4°C for 15 min. The concentration of protein contents was determined with BCA assay kit (Beyotime, Nanjing, China) (Xiong et al., 2017). Eventually western blotting was performed as described above.

### Statistical Analysis

Data were shown as means ± standard error of the mean (SEM). All of the *in vitro* experiments were performed triplicate. Differences between groups were analyzed with one-way analysis of variance (ANOVA), followed by Tukey’s post hoc test using Graphpad prime 5.0 software. A probability (*p*) value of less than 0.05 was considered as statistically significant.

## Results

### ALO Increased Glucose Uptake, Enhanced GLUT4 Expression and Trafficking to the PM in L6 Cells

The effect of ALO on cellular glucose uptake was performed by 2-NBDG assay on different concentrations of ALO (30, 60, and 100 μM) along with both positive (insulin 100 nM) and vehicle control. Results indicated that glucose uptake were significantly enhanced with the increasing of concentration of ALO as compared to the vehicles (Figure 1B). To elucidate whether the enhanced glucose uptake was due to ALO enhanced trafficking to the PM and GLUT4 expression, we measured the intensity of IRAP-mOrange fluorescence and the expression of GLUT4 in L6 cells. We adjusted the dynamics of IRAP-mOrange translocation to reflect the GLUT4 translocation. Following the addition of ALO, a significant increase in IRAP-mOrange fluorescence was observed at the PM (Figures 1C,D). Moreover, we found that insulin and ALO (30, 60, and 100 μM) induced an increase in GLUT4 protein level in L6 cells (Figure 1E). Meanwhile, we measured GLUT4 protein level in PM fraction of L6 cells by western blotting and found that GLUT4 protein level on PM was significantly enhanced in the presence of insulin or ALO (Figure 1F).

### Role of Intracellular Ca^2+^ in ALO-Enhanced GLUT4 Trafficking

**FIGURE 2:**
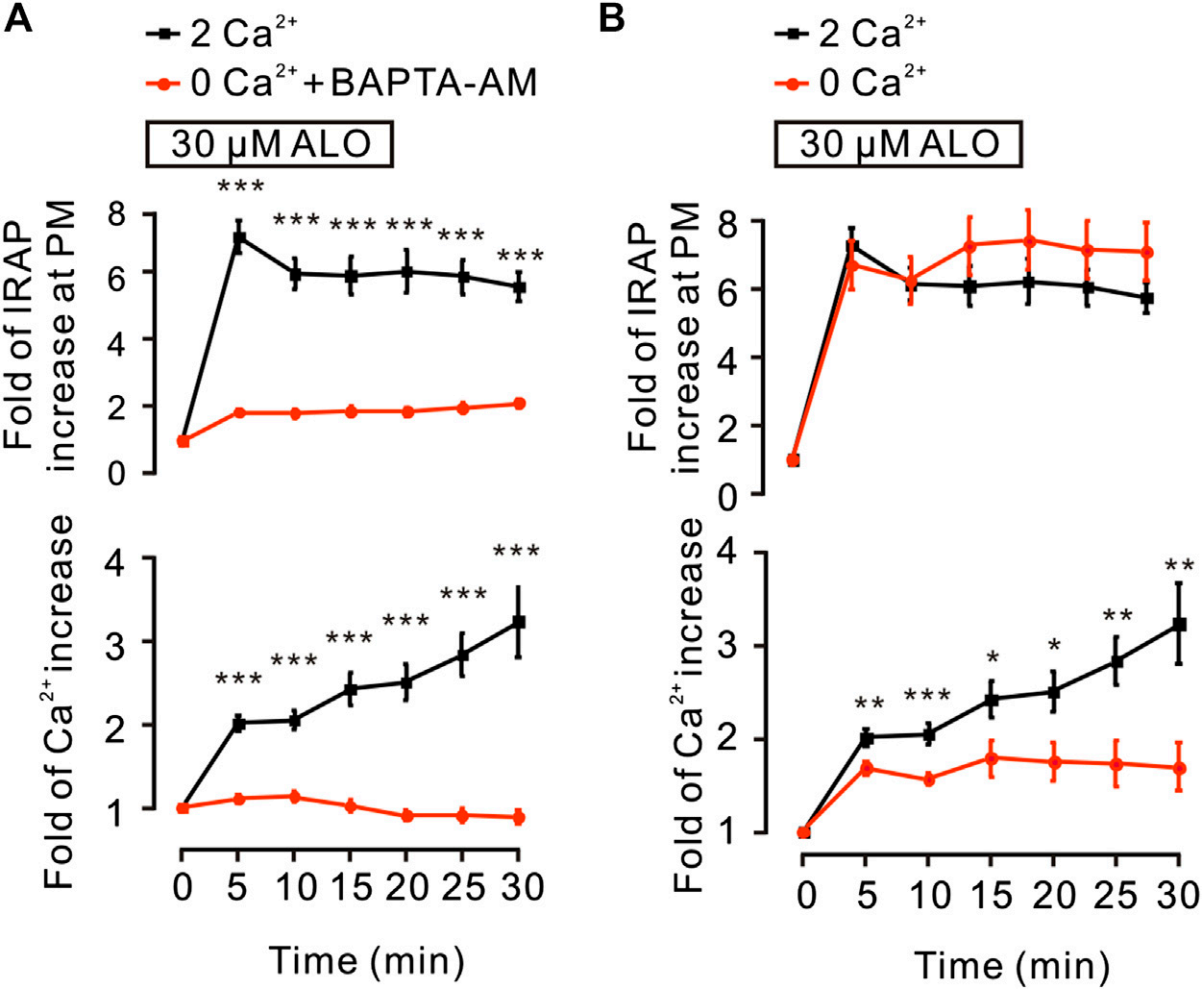
ALO-induced GLUT4 trafficking was related to intracellular calcium. **(A)** ALO-induced IRAP fluorescence increase at the PM was inhibited under 0 mM extracellular Ca^2+^ + 10 *µ*M BAPTA-AM conditions. ***p < 0.001. **(B)** ALO-induced IRAP fluorescence increase at the PM remained unchanged under 0 mM extracellular Ca^2+^ conditions. *p < 0.05; **p < 0.01; ***p < 0.001. Data are means ± SEM (*n* = 30). Two Ca^2+^: PSS containing 2 mM Ca^2+^.

It has been reported that the intracellular Ca^2+^ plays an important role in insulin-induced glucose uptake (Whitehead et al., 2001) and GLUT4 traffic (Deng et al., 2018). Hence, we studied the effects of ALO on intracellular Ca^2+^ and GLUT4 trafficking in L6 cells. Following the addition of ALO, the levels of intracellular Ca^2+^ was increased, while markedly restrained under the conditions of 0 mM Ca^2+^ with 10 *µ*M BAPTA-AM (Figure 2A, *bottom*). Moreover, when cytosolic Ca^2+^ was inhibited, ALO-induced increases of IRAP fluorescence at the PM also was restrained (Figure 2A, *upper*). These data implied that intracellular Ca^2+^ involved in ALO-induced GLUT4 trafficking. Cytosolic Ca^2+^ is composed of intracellular store release and extracellular Ca^2+^ influx. ALO induced increases of intracellular Ca^2+^, which partly decreased following the removal of extracellular Ca^2+^ (Figure 2B, *bottom*). However, ALO-induced increases of IRAP fluorescence at the PM remained unchanged (Figure 2B, *upper*). These results suggested that releasing of intracellular Ca^2+^ is therefore played a key role in ALO-induced GLUT4 trafficking to the PM.

### ALO Enhanced GLUT4 Trafficking and Expression Through PKC and PI3K/Akt Pathways

**FIGURE 3:**
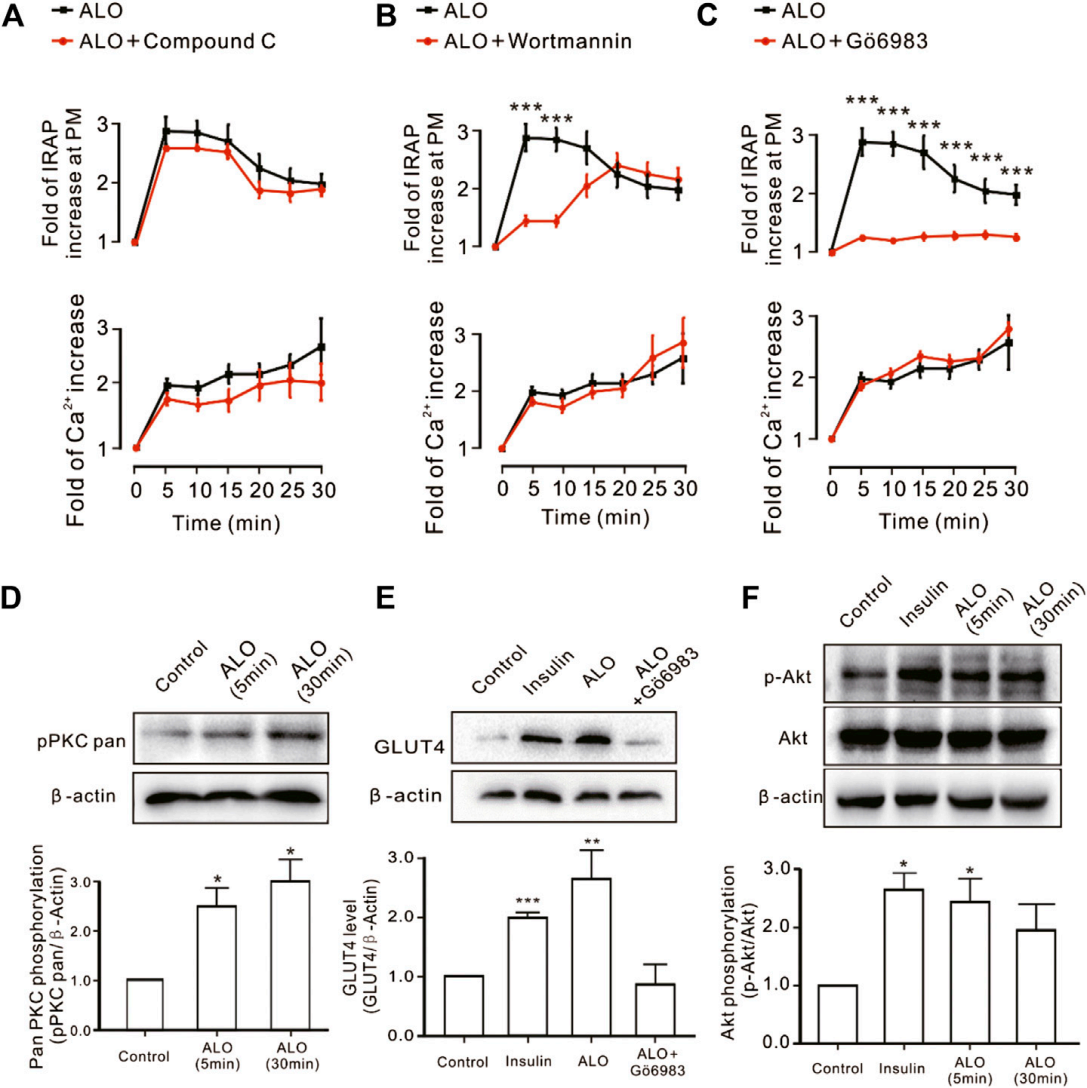
PKC and PI3K/Akt might mediate ALO-induced GLUT4 trafficking and expressing. **(A)** Compound C had no effect on ALO-induced IRAP fluorescence increase at the PM and Ca^2+^ elevation in L6 IRAP-mOrange. **(B)** Wortmannin only inhibited ALO-induced IRAP fluorescence increase at 5 and 10 min. ***p < 0.001. **(C)** Gö6983 strongly inhibited ALO-induced IRAP fluorescence increase. ***p < 0.001. **(D)** ALO induced an increase in PKC phosphorylation level at 5 and 30 min. *p < 0.05. **(E)** Gö6983 inhibited ALO-induced GLUT4 expressing. **p < 0.01; ***p < 0.001. **(F)** ALO only induced an increase in Akt phosphorylation level at 5 min. *p < 0.05 [in **(A–C)** Data are means ± SEM (*n* = 30); in **(D–F)** Data are means ± SEM (*n* = 3)].

We investigated the effect of some signaling pathways associated with GLUT4 trafficking and expression on ALO-enhanced intracellular Ca^2+^ and GLUT4 trafficking to the PM. Compound C is the primary reagent used as an AMPK inhibitor (Laderoute et al., 2010; Chiang et al., 2017), has been used in a few studies. Treating cells with AMPK inhibitor Compound C (10 *µ*M) or PI3K inhibitor Wortmannin (100 nM) or PKC inhibitor Gö6983 (10 *µ*M) for 30 min had no effect on ALO-induced increase of intracellular Ca^2+^ (Figures 3A–C, *bottom*). However, Gö6983 strongly restrained ALO-induced increase of IRAP fluorescence intensity at the PM (Figure 3C, *upper*) and Wortmannin partly inhibited ALO-induced increase of IRAP fluorescence intensity at the PM (Figure 3B, *upper*). On the contrary, Compound C had no effect on ALO-induced increase of IRAP fluorescence intensity at the PM (Figure 3A, *upper*). These data implied that PKC and PI3K/Akt might be involved in ALO-induced GLUT4 trafficking and expression. Thereafter, to validate this observation, we performed western blotting and observed that treating L6 cells with ALO (30 *µ*M) for 5 and 30 min significantly enhanced PKC phosphorylation level (Figure 3D) and the treatment with Gö6983 (10 *µ*M) for 30 min strongly restrained ALO-induced GLUT4 expression (Figure 3E). Interestingly, the addition of ALO only induced an increase in Akt phosphorylation level at 5 min while the rates of increase was reduced at 30 min (Figure 3F). These results indicate that PKC and PI3K/Akt mediate ALO-induced GLUT4 trafficking and expression in L6 cells.

### G Protein and PLC Regulated ALO-Induced Ca^2+^ Increase and GLUT4 Trafficking

**FIGURE 4:**
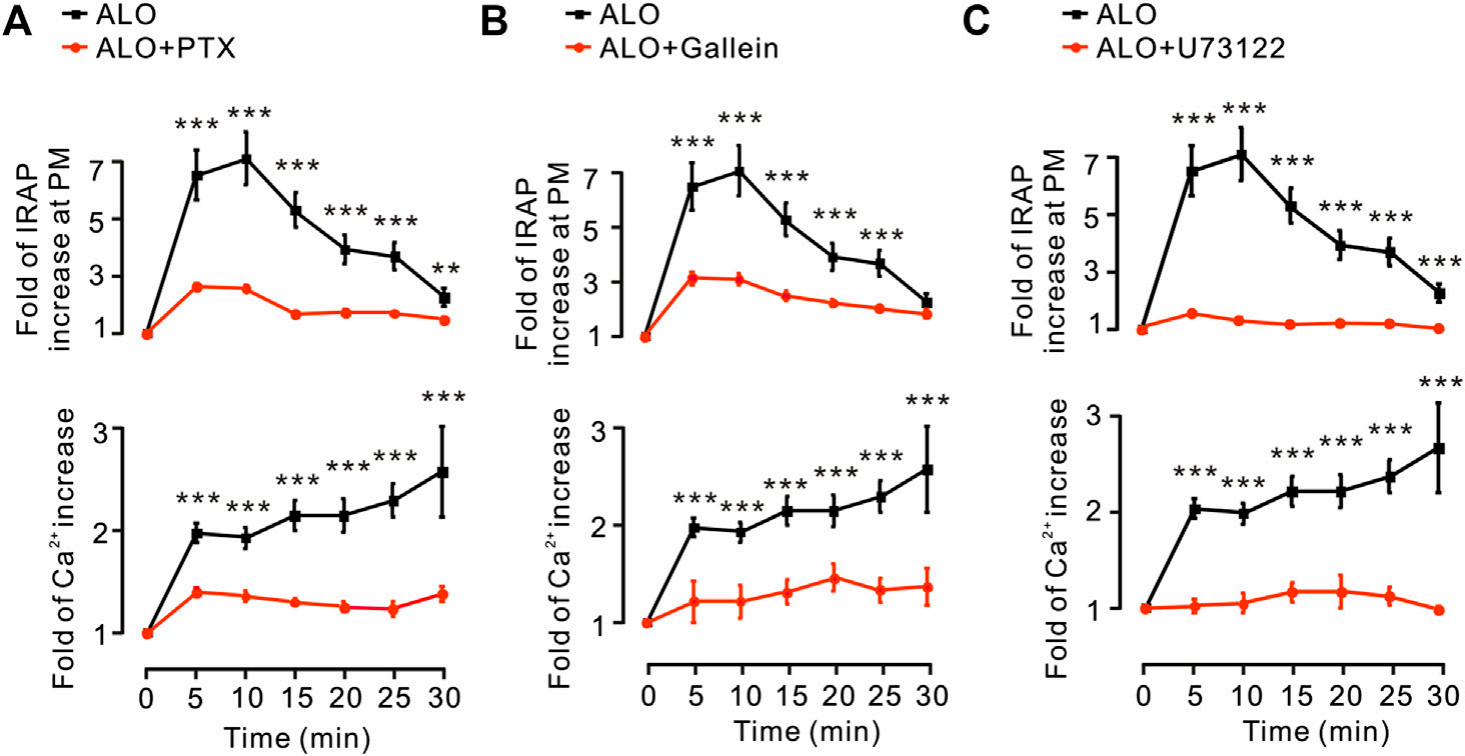
G protein and PLC were involved in ALO-induced Ca^2+^ increase and GLUT4 trafficking. **(A)** PTX inhibited ALO-induced IRAP fluorescence increase at the PM and Ca^2+^ elevation in L6 IRAP-mOrange. **p < 0.01; ***p < 0.001. **(B)** Gallein inhibited ALO-induced IRAP fluorescence increase at the PM and Ca^2+^ elevation in L6 IRAP-mOrange. ***p < 0.001. **(C)** U73122 inhibited ALO-induced IRAP fluorescence increase at the PM and Ca^2+^ elevation in L6 IRAP-mOrange. ***p < 0.001. Data are means ± SEM (*n* = 30).

G protein and PLC are in the upstream of PKC pathway (Diversé-Pierluissi et al., 1997). Thus, we investigated how G protein and PLC regulate ALO-induced Ca^2+^ increase and GLUT4 trafficking. We found that G_α_ protein inhibitor PTX, G_βγ_ protein inhibitor Gallein and PLC inhibitor U73122 were significantly restrained ALO-induced Ca^2+^ increase and GLUT4 trafficking (Figures 4A–C). These results suggested that ALO enhanced GLUT4 trafficking *via* G protein-PLC-PKC signaling pathway.

### IP_3_Rs Was Involved in ALO-Induced GLUT4 Trafficking

**FIGURE 5:**
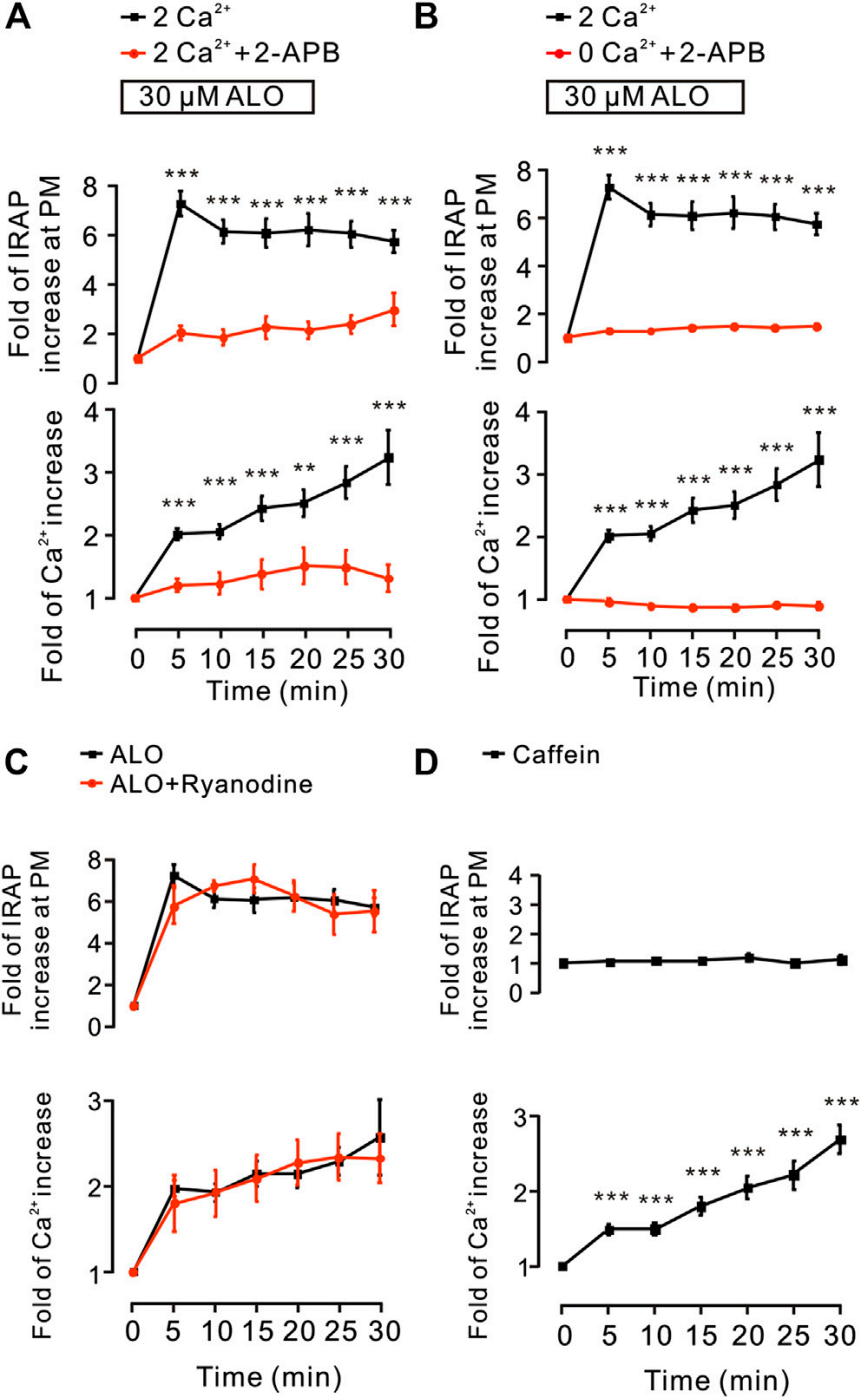
IP_3_Rs was involved in ALO-induced GLUT4 trafficking. **(A)** ALO-induced IRAP fluorescence increase at the PM was inhibited under 2 mM extracellular Ca^2+^ + 100 *µ*M 2-APB conditions. **p < 0.01; ***p < 0.001. **(B)** ALO-induced IRAP fluorescence increase at the PM was inhibited under 0 mM extracellular Ca^2+^ + 100 *µ*M 2-APB conditions. ***p < 0.001. **(C)** ALO-induced IRAP fluorescence increase at the PM and Ca^2+^ increase remained unchanged under 30 *µ*M Ryanodine conditions. **(D)** Caffein (10 mM) induced a Ca^2+^ increase but had no effect on IRAP fluorescence at the PM in L6 IRAP-mOrange. ***p < 0.001. Data are means ± SEM (*n* = 30).

Since these results (Figure 2) suggested that intracellular Ca^2+^ release plays a key role in ALO-induced GLUT4 trafficking to the PM, hence, we used the 2-APB (100 *µ*M), a blocker of IP_3_Rs that regulates intracellular Ca^2+^ release to block the ALO-increased intracellular Ca^2+^. We found that 2-APB significantly inhibited ALO-induced increase of IRAP fluorescence intensity at the PM and intracellular Ca^2+^ under 2 mM (Figure 5A) and 0 mM (Figure 5B) extracellular Ca^2+^ conditions. In addition to IP_3_R, Ryanodine receptor (RyR) is also an important receptor that regulates intracellular calcium release (Pitake and Ochs, 2016). Therefore, we studied the effect of RyR on ALO-induced intracellular Ca^2+^ and GLUT4 trafficking to the PM. We used 30 *µ*M Ryanodine to inhibit RyR, but ALO-induced IRAP fluorescence increase at the PM and intracellular Ca^2+^ increase remained unchanged (Figure 5C). Moreover, the RyR agonist caffeine had no effect on ALO-induced IRAP fluorescence increase at the PM though it enhanced intracellular Ca^2+^ (Figure 5D). Our findings suggested that IP_3_Rs was involved in ALO-increased intracellular Ca^2+^ and GLUT4 trafficking while RyR had no potential effect on it.

### ALO Improved Hyperglycemia and Glucose Tolerance in HFD/STZ-Induced T2DM Rats

**FIGURE 6:**
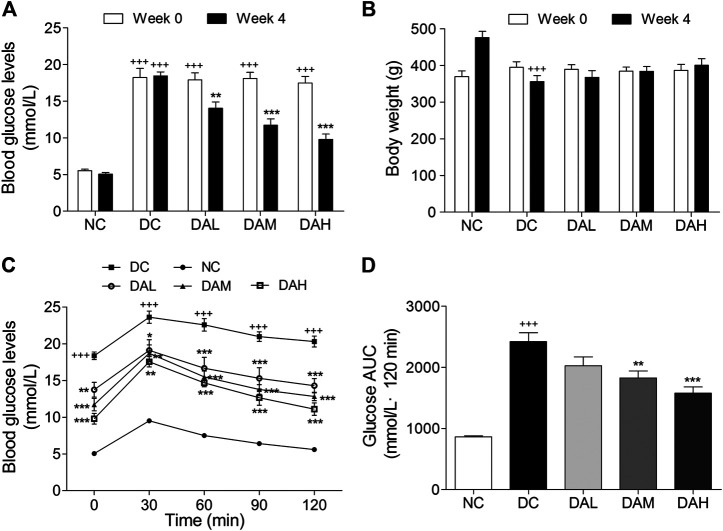
ALO improved hyperglycaemia, body weight loss and glucose tolerance in HFD/STZ-induced T2DM rats. **(A)** FBG levels and **(B)** body weight of rats at 0 and 4 weeks of ALO treatment. **(C)** Blood glucose and **(D)** AUC of glucose during OGTT. Data are means ± SEM (*n* = 8). NC, normal control; DC, diabetic control; DAL, diabetic with low dose ALO of 20 mg/kg; DAM, diabetic with medium dose ALO of 40 mg/kg; DAH, diabetic with high dose ALO of 80 mg/kg. ^+++^ *p* < 0.001, ^++^ *p* < 0.01 vs. NC group, *p < 0.05, **p < 0.01, ***p < 0.001 vs. DC group.

To explore the therapeutic effects of ALO on T2DM, ALO was orally administered to HFD/STZ-induced T2DM rats for four consecutive weeks. Initial FBG levels in T2DM rats were significantly higher than normal rats. T2DM rats exhibited a drastic elevation of FBG levels compared with normal rats, however this elevation was significantly restricted by ALO (Supplementary Figure S2A). At the end of 4 weeks treatment, FBG levels of T2DM rats in ALO-treated groups were significantly reduced as compared with the DC group, whereas normal rats in the NC group maintained stable blood glucose levels during the experiment (Figure 6A). The body weight of T2DM rats was declined from the initiation of treatment, whereas this reduction was effectively reversed by ALO treatment (Supplementary Figure S2B). After 4 weeks of treatment, T2DM rats in the DC group exhibited significant reduction of body weight compared to rats in the NC group, upon treatment with three different doses of ALO, T2DM rats demonstrated different degrees of improvement in their body weight as compared to the DC group (Figure 6B). At the end of the experimental period, an OGTT test was performed. T2DM rats in the DC group showed impaired glucose tolerance, with a sharp increase in blood glucose levels after oral administration of 2.0 g/kg glucose, which remained at a high level over the next 120 min. In contrast, the rise in blood glucose levels in ALO-treated groups was greatly suppressed, and the elevated blood glucose levels were reduced quickly in these rats (Figure 6C). In addition, AUC values of glucose response over the 120 min’ period were calculated. In ALO-treated groups, the AUC of glucose were significantly decreased compared to that in the DC group (Figure 6D). These results suggested that ALO treatment ameliorated body weight disorder, reduced FBG levels and improved the impaired glucose tolerance in T2DM rats.

### ALO Ameliorated Serum Insulin and Lipid Levels in T2DM Rats

**FIGURE 7:**
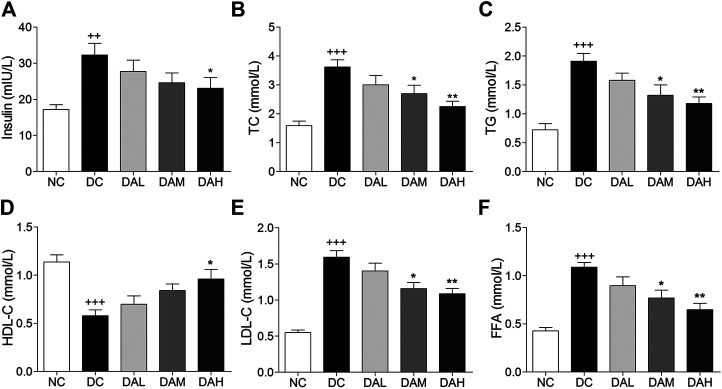
Serum insulin and lipid levels were significantly improved by ALO treatment. **(A)** Effects of ALO on serum insulin level. **(B–F)** Effects of ALO on TC, TG, HDL-C, LDL-C and FFA levels in the serum. Data are means ± SEM (*n* = 8). NC, normal control; DC, diabetic control; DAL, diabetic with low dose ALO of 20 mg/kg; DAM, diabetic with medium dose ALO of 40 mg/kg; DAH, diabetic with high dose ALO of 80 mg/kg. ^+++^ *p* < 0.001, ^++^ *p* < 0.01 vs. NC group, *p < 0.05, **p < 0.01 vs. DC group.

T2DM rats exhibited much higher insulin levels than normal rats. After 4 weeks of treatment with ALO, serum insulin levels were clearly reduced compared with those in the DC group (Figure 7A). Significant disorders of lipid metabolism were observed in T2DM rats, including a marked increase of TC, TG, FFA and LDL-C, and decline of HDL-C in blood serum as compared to normal rats. Following 4 weeks of treatment with ALO, serum TC, TG, FFA and LDL-C levels were significantly decreased and HDL-C level was markedly enhanced compared with the DC group (Figures 7B–F).

### ALO Alleviated Hepatic Steatosis in T2DM Rats

**FIGURE 8:**
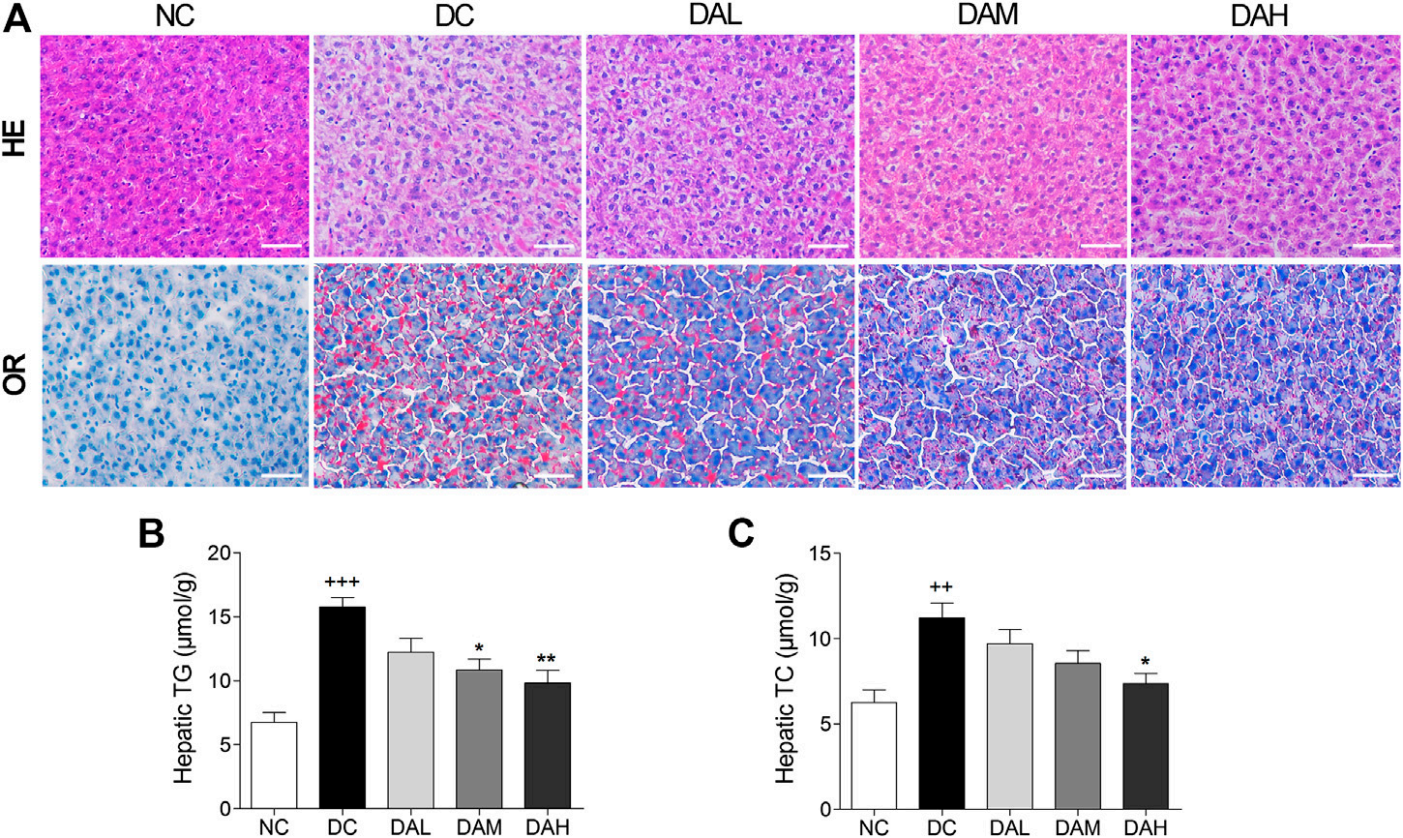
ALO treatment prevented hepatic steatosis in T2DM rats. **(A)** HE and OR staining of the liver from T2DM rats. **(B–C)** The contents of hepatic TG and TC from T2DM rats. Scale bar, 50 μm. Data are means ± SEM (*n* = 8). TG, triglyceride; TC, total cholesterol; NC, normal control; DC, diabetic control; DAL, diabetic with low dose ALO of 20 mg/kg; DAM, diabetic with medium dose ALO of 40 mg/kg; DAH, diabetic with high dose ALO of 80 mg/kg. ^+++^ *p* < 0.001, ^++^ *p* < 0.01 vs. NC group, *p < 0.05, **p < 0.01 vs. DC group.

Morphologic analysis showed that T2DM rats in the DC group had significant hepatic steatosis, whereas normal rats in the NC group showed normal cell morphology. By contrast, ALO treatment decreased the degree of hepatic steatosis and smaller fat droplets were observed in individual hepatocyte, especially in the DAH group (Figure 8A, panel HE). We also examined hepatic lipid by Oil red O staining, and found ALO treatment suppressed accumulation of lipid (Figure 8A, panel OR). Additionally, the contents of TC, TG in liver were alleviated with ALO treatment (Figures 8B,C).

### ALO Protected Pancreatic Islet Function in T2DM Rats

**FIGURE 9:**
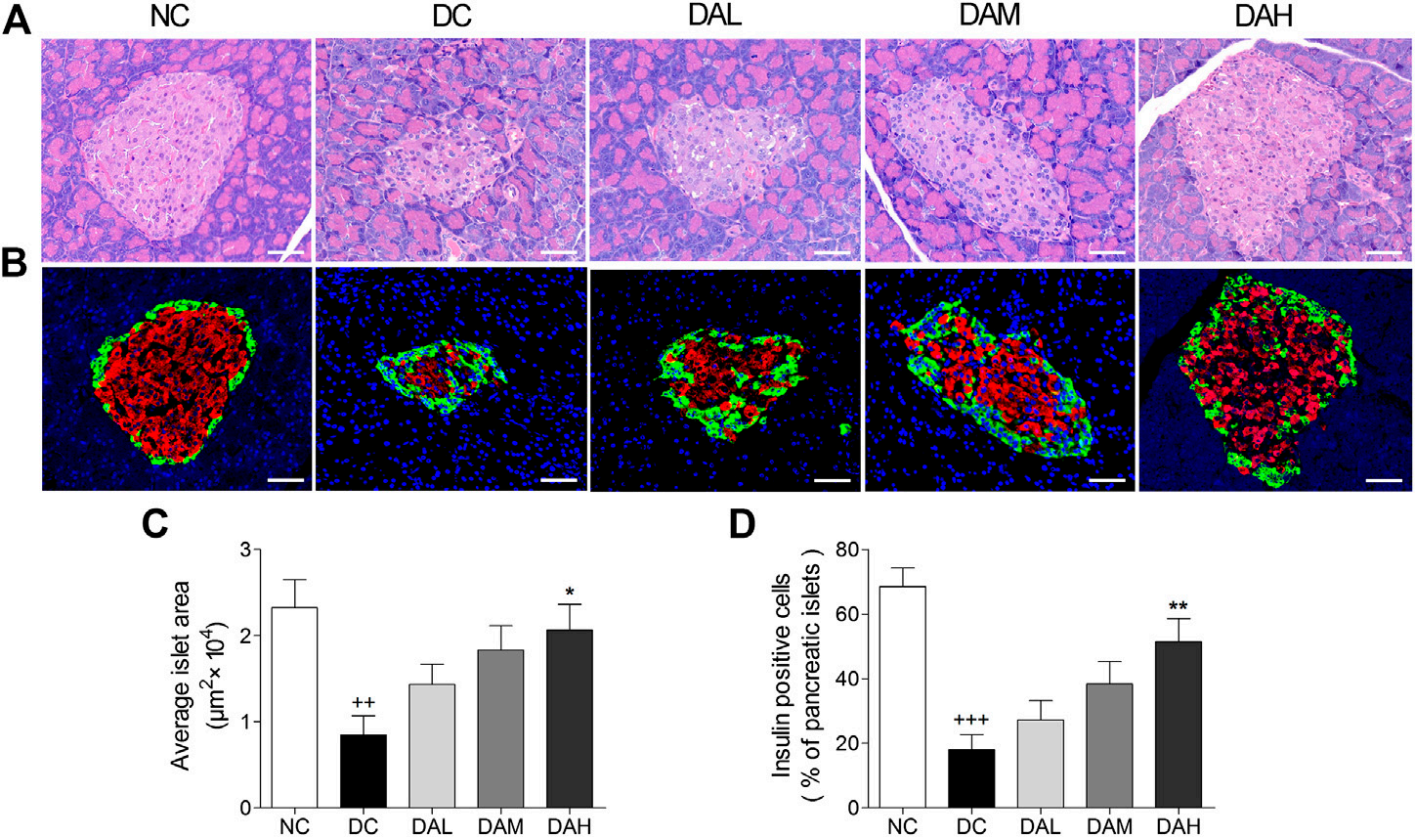
ALO treatment protected pancreatic islet function in T2DM rats. **(A)** Pancreas sections were stained with hematoxylin-eosin and observed under light microscopy. **(B)** Pancreas sections were stained with insulin (red), glucagon (green) and DAPI (blue), observed under fluorescence microscopy. Scale bar, 50 μm. **(C)** Quantified average islet area and **(D)** the percentage of insulin-positive cells in pancreatic islets. Data are means ± SEM. *n* = 4 rats per group. NC, normal control; DC, diabetic control; DAL, diabetic with low dose ALO of 20 mg/kg; DAM, diabetic with medium dose ALO of 40 mg/kg; DAH, diabetic with high dose ALO of 80 mg/kg. ^+++^ *p* < 0.001, ^++^ *p* < 0.01 vs. NC group, *p < 0.05, **p < 0.01 vs. DC group.

HE staining of pancreatic islets in the NC group showed the normal islet morphology. The islet structure was severely damaged in T2DM rats, whereas ALO treatment exhibited a protective effect on islet morphology (Figure 9A). Besides, average islet area in T2DM rats was significantly decreased in comparison with normal rats. In ALO-treated groups, those decreases were largely alleviated (Figure 9C). Immunofluorescence staining showed that the insulin-positive beta cell area in normal rats occupied a majority of the islet area, whereas staining was drastically diminished in T2DM rats. ALO treatment prominently ameliorated this destruction and produced a similar morphology to normal islets. More insulin and less glucagon staining were observed in T2DM rats of ALO-treated groups compared with the DC group (Figure 9B). Consistent with these results, treatment with ALO significantly enhanced the percentage of insulin-positive cells in pancreatic islets (Figure 9D). These results suggested that ALO treatment protected pancreatic beta cells in T2DM rats.

### ALO Activated PKC and Akt Phosphorylation and Enhanced GLUT4 Expression in Insulin Target Tissues

**FIGURE 10:**
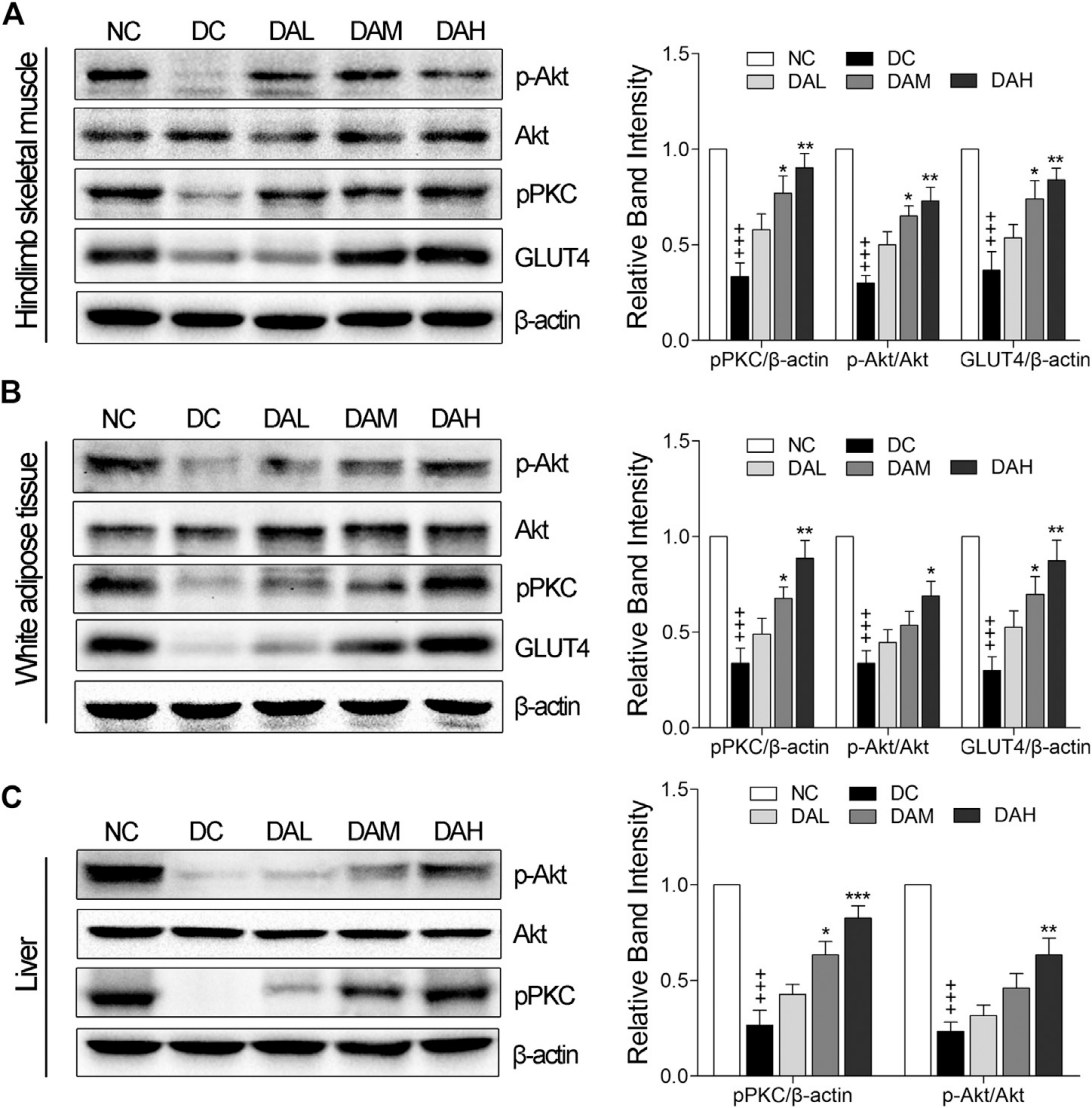
ALO enhanced GLUT4 expression, activated PKC and Akt phosphorylation in insulin target tissues. Western blot analysis of PKC and Akt phosphorylation, GLUT4 expression in **(A)** skeletal muscle and **(B)** WAT of T2DM rats. **(C)** Western blot analysis of PKC and Akt phosphorylation in liver of T2DM rats. NC, normal control; DC, diabetic control; DAL, diabetic with low dose ALO of 20 mg/kg; DAM, diabetic with medium dose ALO of 40 mg/kg; DAH, diabetic with high dose ALO of 80 mg/kg. Data are mean ± SEM, shown as relative band intensity compared with NC group. ^+++^ *p* < 0.01 vs. NC group, *p < 0.05, **p < 0.01, ***p < 0.001 vs. DC group.

We examined *in vivo* expression of GLUT4, p-PKC (pan) and p-Akt (phosphorylation of Akt) in skeletal muscle and WAT. The results showed that the treatment with ALO increased GLUT4 expression, phosphorylation of both PKC and Akt in skeletal muscle and WAT of T2DM rats. Especially, ALO was more effective in phosphorylating PKC than Akt (Figures 10A,B). We also observed the phosphorylation of Akt and PKC, and not GLUT4, in the liver using western blotting. Levels of p-PKC and p-Akt were significantly enhanced in the livers of T2DM rats treated with ALO (Figure 10C).

## Discussion

Some traditional Chinese herbs have been used to treat diabetes mellitus in some Chinese ethnic hospitals for a long time and showed promising clinical therapeutic effect (Li et al., 2004). As a main alkaloidal principle of the traditional Chinese ethnic medicine *S. alopecuroides* L., previous studies have shown that ALO has many significant biological activities. Nonetheless, the effect of ALO is unclear in the treatment of diabetes. We discovered that ALO has beneficial effects in promoting GLUT4 translocation and glucose uptake *in vitro*, indicating it has the potential to relieve diabetes.

Previous studies reported that GLUT4 is a glucose transporter expressed primarily in adipose and muscle tissues, and a key regulator of whole-body glucose homeostasis (Carvalho et al., 2005). Increasing GLUT4 expression and translocation to the cell membrane of peripheral insulin target tissues are responsible for improvements in insulin sensitivity. Therefore, we mainly studied the hypoglycemic activity of ALO with GLUT4 as the target and related mechanism. In the present study, following the addition of ALO, glucose uptake in L6 cells was promoted in a dose-dependent manner (Figure 1B). and a significant increase in IRAP-mOrange fluorescence was observed at the PM (Figures 1C,D), the GLUT4 protein level in the cell as wells as on the plasma membrane exhibited significant upward trends (Figures 1E–F). These results demonstrated the effect of ALO on promoting GLUT4 activity, thereby increasing glucose uptake.

AMPK (Huang et al., 2016), PI3K/Akt (Ramachandran and Saravanan, 2015) and PKC (Tsuchiya et al., 2013) pathways are involved in GLUT4 translocation and expression. Some natural products can increase GLUT4 translocation and expression through these three pathways to relieve the T2DM. For example, carnosol increases skeletal muscle cell glucose uptake *via* AMPK-dependent GLUT4 translocation (Vlavcheski et al., 2018), sanggua drink increase GLUT4 gene and protein expressions level in T2DM rats *via* PI3K/AKT pathway (Cai et al., 2018) and diacylglycerol promotes GLUT4 translocation *via* activation of PKCε in adipocytes (Tsuchiya et al., 2013). Imaging experiments along with western blotting analysis substantiated that ALO enhanced GLUT4 translocation and expression *via* PI3K/Akt and PKC pathways, but AMPK pathway didn’t involved in (Figure 3). Interestingly, Wortmannin partly restrained ALO-enhanced GLUT4 translocation, but the GLUT4 translocation recovered to near control group levels in 20 min (Figure 3B, *upper*). Moreover, the addition of ALO only induced an increase in Akt phosphorylation level at 5 min while the increase was reduced at 30 min (Figure 3F). We hypothesized that the recovery of GLUT4 translocation might be due to the compensation of PKC pathway when the PI3K/Akt pathway was inhibited and ALO-increased GLUT4 translocation might have certain timeliness *via* PI3K/Akt pathway. Our data showed that PKC pathway is vital for ALO-induced GLUT4 translocation and expression. Earlier studies reported that G protein and PLC were in the upstream of PKC pathway (Diversé-Pierluissi et al., 1997; Reed et al., 2000). Treatment with the G_β/γ_ protein blocker Gallein, G_α_ protein blocker PTX and PLC blocker U73122 were shown to significantly block ALO-induced GLUT4 translocation (Figure 4, *upper*). Therefore, we concluded that ALO enhanced GLUT4 translocation *via* G protein-PLC-PKC signaling pathway in L6 cells.

**FIGURE 11:**
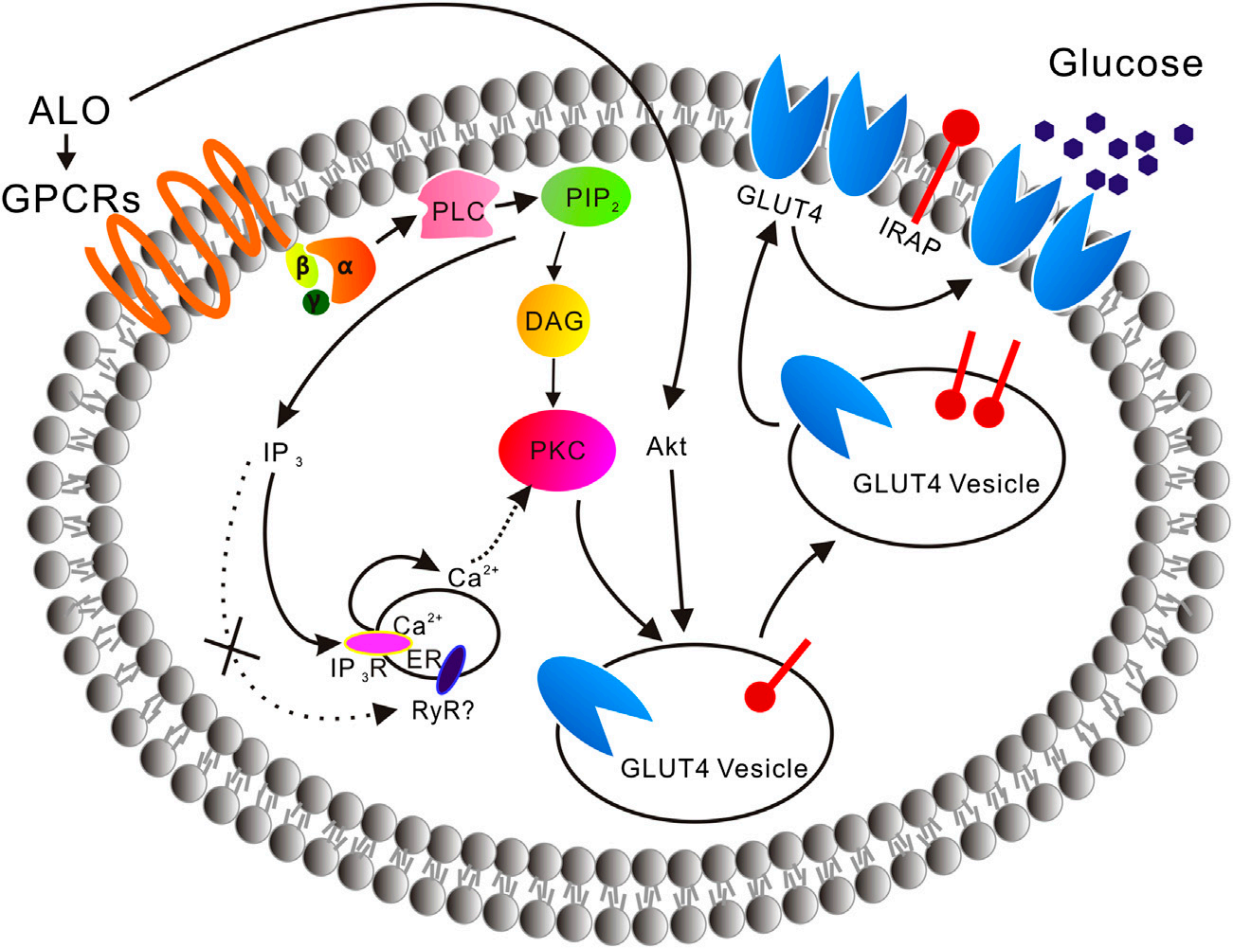
A model is proposed of the ALO-induced increase of glucose uptake. ALO *via* G protein-PLC-PKC pathway and PI3K/Akt pathway enhances GLUT4 expression and translocation to the PM, thus increasing glucose uptake. ALO-increased intracellular Ca^2+^ may be involved in activating the PKC *via* G protein-PLC-IP_3_R-Ca^2+^ pathway.

In the absence of extracellular Ca^2+^, the translocation of GLUT4 caused by ALO stimulation was not affected, but only partially affected the increase of intracellular calcium ion concentration, indicating that Ca^2+^ release played a key role in ALO-induced GLUT4 translocation (Figure 2). After that we aim to clarify how exactly Ca^2+^ regulated ALO-induced GLUT4 translocation in L6 cells. IP_3_Rs and RyR are important receptors that regulate intracellular Ca^2+^ release. We found that IP_3_Rs was involved in ALO-increased intracellular Ca^2+^ and GLUT4 translocation, however, Ryanodine didn't affect ALO effect (Figure 5). Besides our data showed that PTX, Gallein and U73122 significantly restrained ALO-increased intracellular Ca^2+^ (Figure 4, *bottom*). Hence, ALO could increase the intracellular Ca^2+^ *via* G protein-PLC-IP_3_R-Ca^2+^ pathway. In summary, *in vitro* studies demonstrated that ALO-induced enhancement of GLUT4 expression and translocation are mediated by G protein-PLC-PKC and PI3K/Akt pathways, thus increasing glucose uptake. Recent study showed that raising intracellular cytosolic Ca^2+^ concentration can activate PKCs which promote the gain of surface GLUT4 level (Deng et al., 2018). Data obtained herein are concurrence with this previous study, ALO-increased intracellular Ca^2+^ might be involved in activating the PKC *via* G protein-PLC-IP_3_R-Ca^2+^ pathway (Figure 11).

To investigate the possible antidiabetic effects and potential mechanisms of ALO *in vivo,* a T2DM rat model was evaluated in this study. Long term HFD would result in insulin resistance and hyperinsulinaemia, and under the strain of compensatory hyperinsulinaemia, beta-cells could be easily damaged by low-dose of STZ (Kusakabe et al., 2009). HFD/STZ-induced diabetic rats exhibited hyperglycemia, insulin resistance, hyperlipidemia, impaired glucose tolerance and signaling activity, which would closely mimic the metabolic characteristics of human T2DM (Reed et al., 2000). In the present study, ALO treatment significantly reduced FBG levels, improved body weight from being lost and glucose intolerance in T2DM rats (Figure 6), indicating that treatment with ALO can improve glucose homeostasis. Furthermore, the concomitant decrease in serum insulin level (Figure 7A) and improvement of glucose tolerance suggested that ALO is helpful to enhance insulin sensitivity and relieve insulin resistance. The histopathology observation of pancreas showed ALO had protective effects on pancreatic beta-cells in T2DM rats (Figure 9).

Lipid metabolism disorders including obesity, hyperlipidemia, and non-alcoholic fatty liver disease are always companied with T2DM (Adiels et al., 2008). TC and TG accumulation in the liver contributed to enhanced risk of insulin resistance and also led to the pathogenesis of fatty liver (Jiao et al., 2008). Indices of blood lipid metabolism (such as HDL-C, LDL-C, FFA, TG, and TC) were significantly improved (Figure 7), as well as the contents of hepatic TC and TG were also significantly reduced by ALO treatment (Figures 8B,C). In addition, histopathology observation of liver revealed that ALO reduced hypertrophy of hepatocytes and suppressed lipid accumulation (Figure 8A). These results indicated that ALO was capable to control the hyperlipidemia and hepatic steatosis in T2DM rats. To investigate the molecular mechanism underlying the effects of ALO on T2DM, proteins from insulin target tissues (primarily skeletal muscle, adipose tissues and liver) were analyzed by western blotting assays, and the results revealed that ALO increased the expression of GLUT4 and activated the phosphorylation of PKC and Akt in insulin target tissues of T2DM rats (Figure 10). It can be claimed that the ALO activates PKC and Akt phosphorylation and promotes GLUT4 expression, thus facilitating glucose utilization to ameliorate insulin resistance in T2DM.

## Conclusion

ALO, a quinolizidine alkaloid isolated from *S. alopecuroides* L., which used in the traditional Uygur medicine exhibited significant effects in promoting glucose uptake *via* targeting GLUT4. ALO *via* G protein-PLC-PKC pathway and PI3K/Akt pathway enhance GLUT4 expression and translocation to the PM, thus increasing glucose uptake. ALO-increased intracellular Ca^2+^ may be involved in activating PKC *via* G protein-PLC-IP_3_R-Ca^2+^ pathway. *In vivo* administration of ALO to diabetic rats demonstrated its effective use against T2DM. We report for the first time that ALO has great potentiality to be exploited as a hypoglycemic agent which is validated in this study through *in vivo* and *in vitro* studies.

## Data Availability Statement

The raw data supporting the conclusions of this article will be made available by the authors, without undue reservation, to any qualified researcher.

## Ethics Statement

The animal study was reviewed and approved by the university animal ethics committee (Approval Number: S08916110D).

## Author Contributions

PZ and XY: contributed to the conception of the study; GS, YH, MX, and ZY: performed the experiments and analyzed the data; GS, YH, and MX: drafted the manuscript; QL and JS: assisted with the experiments; PZ and XY: revised the manuscript. All authors approved the final version of the manuscript and agree to be accountable for all aspects of the work.

## Funding

The present study was financially supported by National Natural Science Foundation of China grants (81573561, 81774000, and 31070744), Fundamental Research Funds for the Central Universities, South-Central University for Nationalities (CZY20004), Wuhan Applied Basic Research Program of Science and Technology (2017060201010217) and Fund for Key Laboratory Construction of Hubei Province (2018BFC360).

## Conflict of Interest

The authors declare that the research was conducted in the absence of any commercial or financial relationships that could be construed as a potential conflict of interest.

## Abbreviations

ALO, Aloperine; AMPK, (AMP)-activated protein kinase; FBG, Fasting blood glucose; FFA, Free fatty acid; GLUT4, Glucose transporter 4; GSVs, GLUT4 storage vesicles; HFD, High-fat diet; HDL-C, High density lipoprotein cholesterol; IR, Insulin resistance; IRAP, Insulin-regulated aminopeptidase; LDL-C, Low density lipoprotein cholesterol; OGTT, Oral glucose tolerance test; p-AMPKα, Phosphorylation of the (AMP)-activated protein kinase; PKB (Akt), Protein kinase B; PKC, Protein kinase C; STZ, Streptozotocin; TC, Total cholesterol; TG, Triglycerides; T2DM, Type 2 diabetes mellitus; WAT, White adipose tissue.
