# Supplementary material for: Aloperine Relieves Type 2 Diabetes Mellitus via Enhancing GLUT4 Expression and Translocation
Source: Front Pharmacol. 2021 Jan 25;11:561956. doi: 10.3389/fphar.2020.561956 (PMC7868325; doi:10.3389/fphar.2020.561956)
Supplement: Supplementary file 1 [file datasheet1.doc]

Supplementary Material

**S1. ^1^H-NMR, ^13^C-NMR and EIMS spectrum of Aloperine**


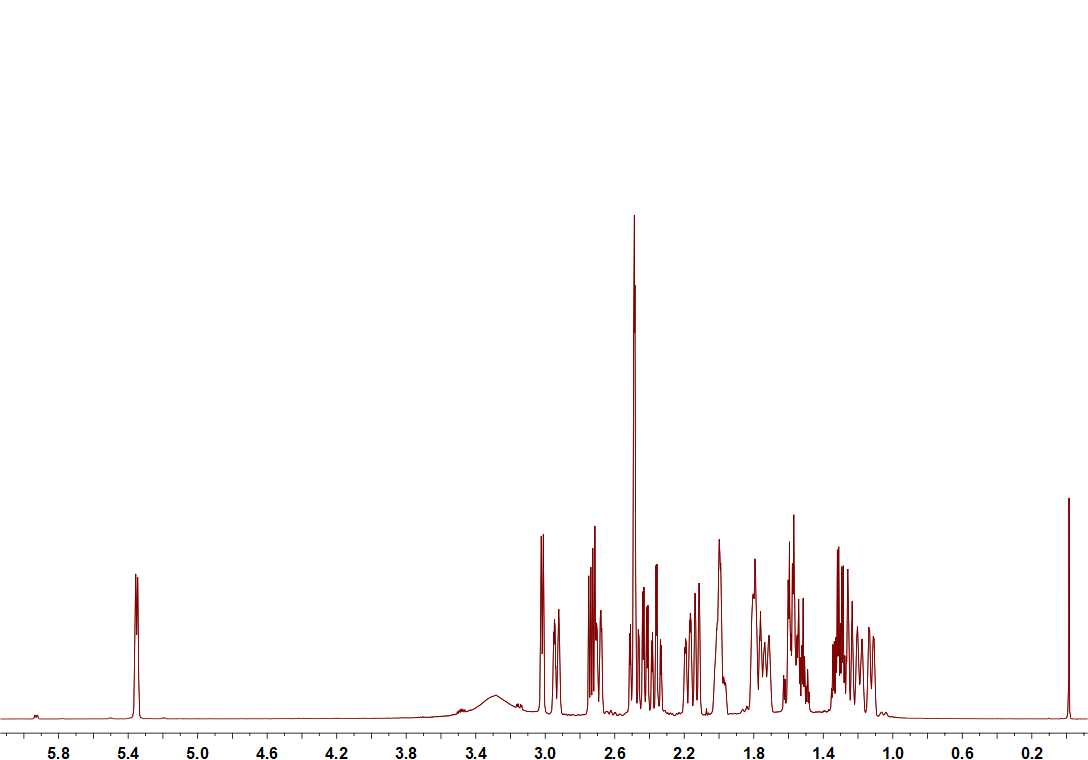


**^1^H-NMR spectrum of Aloperine**

**
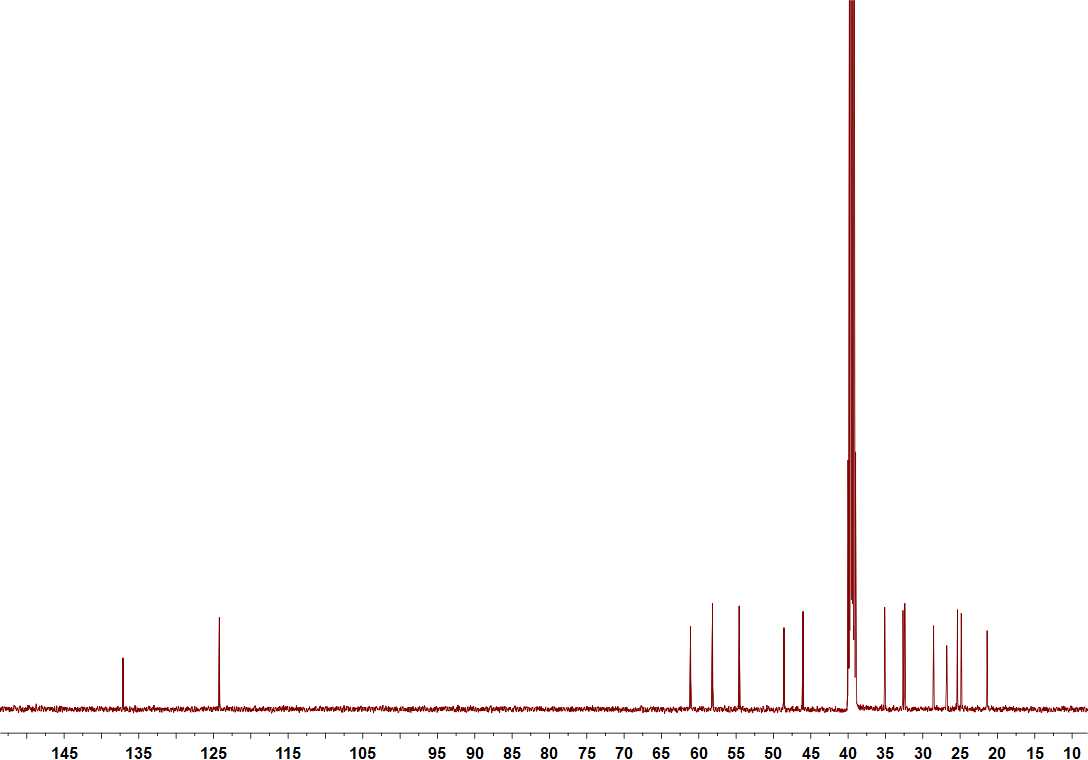
**

**^13^C-NMR spectrum of Aloperine**

**EIMS spectrum of Aloperine**

**S2. The dynamic changes of FBG levels and body weight**


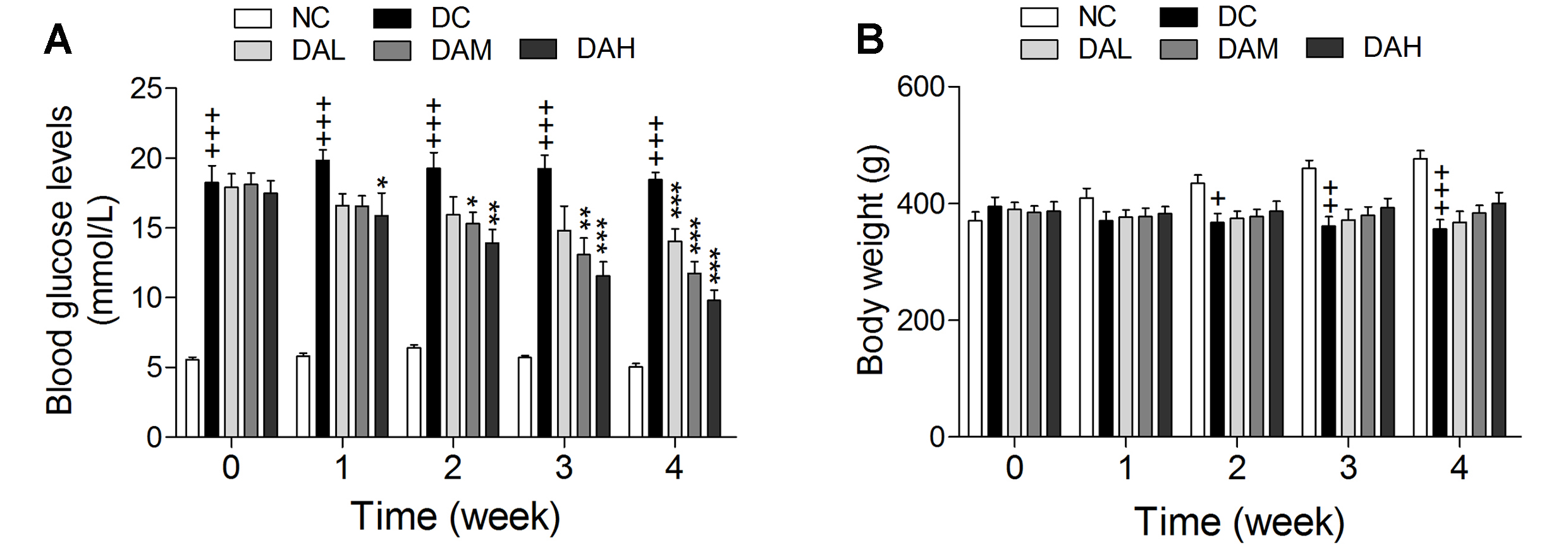


**Supplementary Figure S2**. The dynamic changes of FBG levels (A) and body weight (B) of T2D rats during 4 weeks of treatment with ALO. Data are means ± SEM (n = 8). ^+^P < 0.05, ^++^P < 0.01, ^+++^P < 0.001 versus NC group, ^*^P < 0.05, ^**^P < 0.01, ^***^P < 0.001 versus DC group.
